# The *Saccharomyces boulardii* CNCM I-745 Strain Shows Protective Effects against the *B. anthracis* LT Toxin

**DOI:** 10.3390/toxins7114455

**Published:** 2015-10-30

**Authors:** Rodolphe Pontier-Bres, Patrick Rampal, Jean-François Peyron, Patrick Munro, Emmanuel Lemichez, Dorota Czerucka

**Affiliations:** 1 Centre Scientifique de Monaco, Monaco 98000, Monaco; E-Mails: rpontier-bres@centrescientifique.mc (R.P.-B.); rampal@chpg.mc (P.R.); 2 Team Inflammation, Cancer, Cancer Stem Cells, Centre Méditerranéen de Médecine Moléculaire (C3M), INSERM, U1065, Nice 06204, France; E-Mail: jean-francois.peyron@unice.fr; 3 Faculté de Médecine, UFR Médecine, IFR50, Université de Nice-Sophia Antipolis, UNSA, Nice 06204, France; E-Mails: patrick.munro@unice.fr (P.M.); emmanuel.lemichez@unice.fr (E.L.); 4 Team Microbial Toxins in Host Pathogen Interactions, Centre Méditerranéen de Médecine Moléculaire (C3M), INSERM, U1065, Nice 06204, France

**Keywords:** anthrax toxins, probiotics, *Saccharomyces boulardii*

## Abstract

The probiotic yeast *Saccharomyces boulardii* (*S. boulardii*) has been prescribed for the prophylaxis and treatment of several infectious diarrheal diseases. Gastrointestinal anthrax causes fatal systemic disease. In the present study, we investigated the protective effects conferred by *Saccharomyces boulardii* CNCM I-745 strain on polarized T84 columnar epithelial cells intoxicated by the lethal toxin (LT) of *Bacillus anthracis*. Exposure of polarized T84 cells to LT affected cell monolayer integrity, modified the morphology of tight junctions and induced the formation of actin stress fibers. Overnight treatment of cells with *S. boulardii* before incubation with LT maintained the integrity of the monolayers, prevented morphological modification of tight junctions, restricted the effects of LT on actin remodeling and delayed LT-induced MEK-2 cleavage. Mechanistically, we demonstrated that in the presence of *S. boulardii*, the medium is depleted of both LF and PA sub-units of LT and the appearance of a cleaved form of PA. Our study highlights the potential of the *S. boulardii* CNCM I-745 strain as a prophylactic agent against the gastrointestinal form of anthrax.

## 1. Introduction

*Bacillus anthracis*, the causative agent of anthrax, is a large, Gram-positive, rod-shaped, aerobic, spore-forming bacterial pathogen. *B*. *anthracis* can infect a large variety of hosts by cutaneous, gastrointestinal, and pulmonary (inhalation) routes. The infection progresses into a fatal systemic disease [1,2,3]. The easily diagnosed cutaneous form presents as a small pimple that develops into a painless black eschar characteristic of anthrax. Gastrointestinal anthrax is typically related to the ingestion of a *B. anthracis*-contaminated meal. There are two forms of gastrointestinal anthrax: oropharyngeal and intestinal. In the intestinal form, anthrax spore deposits can cause ulcerative lesions anywhere from the jejunum to the cecum [4]. In the gastrointestinal and inhalation forms, the illness is insidious at first, with mild symptoms of gastroenteritis [2,5]. Early diagnosis is difficult, and the disease abruptly develops into its systemic form, rapidly becoming resistant to treatment.

*B. anthracis* synthesizes a three-component toxin as major virulence factor [5,6,7]. Heptamers/octamers of the protective-antigen (PA) bind to receptors on the host cell and associate with the lethal factor (LF, 90 kDa) to generate a lethal toxin (LT for PA + LF) and/or with the edema factor (EF, 89 kDa) to form an edema toxin (ET for PA + EF). EF is a calmodulin-dependent adenylate cyclase that increases the intracellular cAMP concentration [8]. LF is a zinc-dependent metalloprotease that specifically cleaves the *N*-terminus of most mitogen-activated protein kinase kinases (MAPKK or MEK) and NLRP1B, a key component of the inflammasome system [9,10]. Inhibition of MEKs by LT produces various cytotoxic effects, notably the recently described induction of actin cytoskeleton remodeling [7,11,12,13,14]. The mechanisms of cell penetration by the toxins have been thoroughly elucidated over the past twenty years and are distinguished by three major steps: receptor binding, internalization, and translocation of enzymatic factors across endosomal membranes. PA_83_ binds to a specific cell receptor (ANTXR1 or TEM8 (tumor endothelial marker-8) and ANTXR2 or CMG2 (capillary morphogenesis gene-2)), allowing its cleavage by an extracellular furin protease to a 63-kDa form (PA_63_) [7].

The nonpathogenic yeast *Saccharomyces boulardii* (*S. boulardii*) has been prescribed for the past 30 years for prophylaxis and treatment of diarrheal diseases caused by bacteria. Clinical evidence of the efficacy of this probiotic yeast was recently reviewed by McFarland [15]. Experimental studies demonstrated that *S. boulardii* has an arsenal of anti-pathogenic strategies that could be classified into three principal areas: luminal action, trophic action and mucosal anti-inflammatory action. In a toxin-mediated disease, *S. boulardii* may act in the intestinal lumen by blocking the receptor or by the direct destruction of the pathogenic toxin. *S. boulardii* has been shown to synthesize several proteases: a 54-kDa serine protease that directly degrades toxins A and B from *C. difficile* and their receptors [16,17,18] and a 63-kDa phosphatase that destroys the endotoxin of pathogenic *E. coli* [19]. Toxin adhesion to the yeast cell wall has been reported for cholera toxin (CT) [20]. Finally, *S. boulardii* has been shown to interfere with several signaling pathways activated by bacterial toxins and implicated in cellular responses to the infection: (i) *S. boulardii* decreased adenylate cyclase activation and consequently chloride secretion induced by CT [21,22]; (ii) *S. boulardii* decreased the activation of ERK1/2 mitogen-activated protein kinase and consequently IL-8 secretion induced by *C. difficile* toxin A [23]; and (iii) *S. boulardii* reversed the drop in the intestinal permeability of the human colonic mucosa after exposure to toxins A and B from *C. difficile* [17].

These anti-toxin activities of *S. boulardii* prompted us to investigate the effect of this yeast against PA and LF factors produced by *Bacillus anthracis*.

## 2. Results

### 2.1. S. boulardii Protects against Cell Intoxication by LT

**Figure 1:**
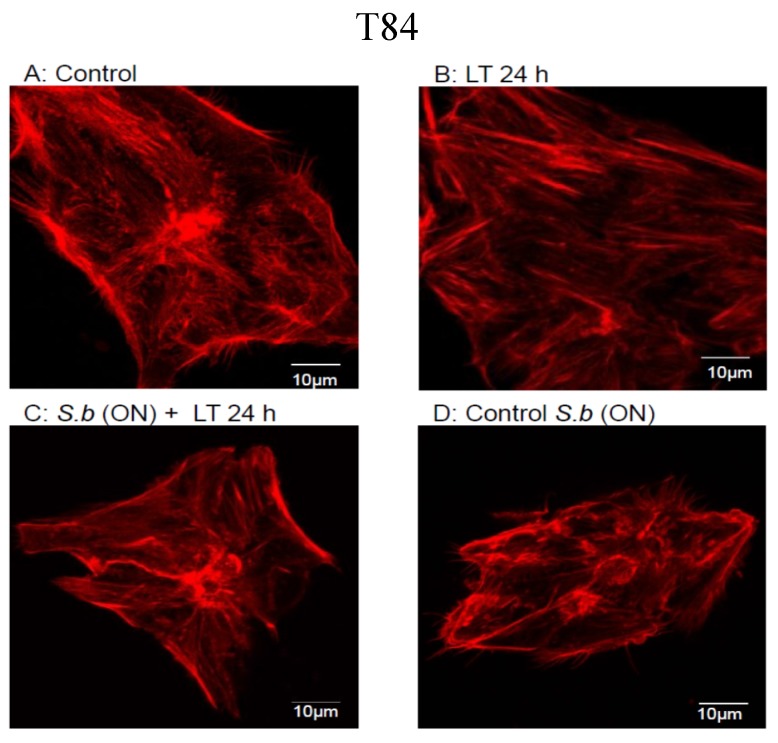

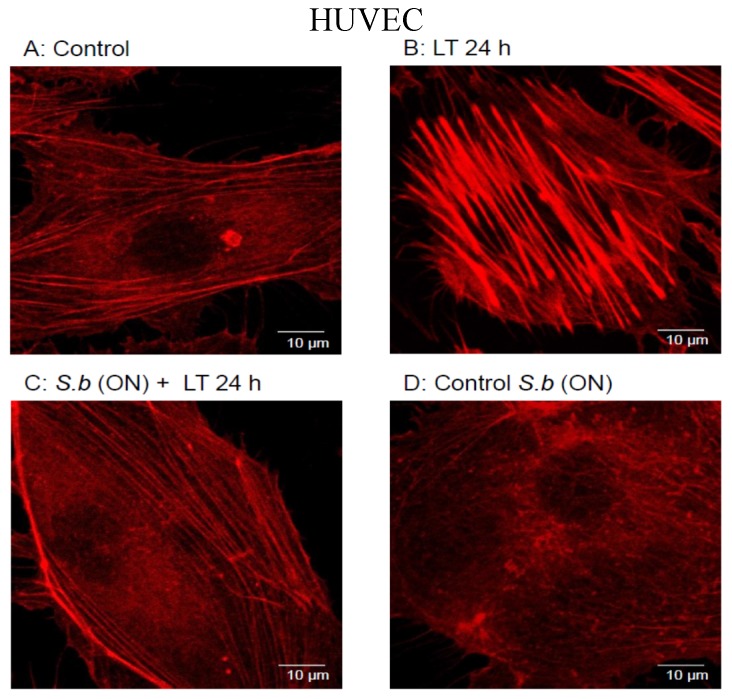
Actin cytoskeleton organization in T84 cells (**upper**) and HUVECs (**down**). Pictures show control cells (**A**); cells exposed to LT for 24 h (LT 24 h) (**B**); cells incubated with *S. boulardii* (ON*)* before LT addition for 24 h (**C**); and cells incubated with *S. boulardii* overnight (ON) as sole treatment (**D**). TRITC-conjugated phalloidin was used for F-actin labeling. Identical results were obtained at least in three independent experiments. Bar = 10 μm.

To evaluate inhibitors of the intoxication process, we took advantage of the massive actin cytoskeleton reorganization promoted by LT through MEK inhibition [24,25]. To begin, we tested whether *S. boulardii* treatment blocked cell intoxication due to LT. We tested the effect of the LT toxin on the actin cytoskeleton in T84 cells. Polarized monolayers of T84 cells were apically exposed to the LT toxin for 24 h, and the formation of stress cables was monitored by confocal microscopy. After 24 h of intoxication, LT induced stress fiber formation in the T84 cells compared with control cells (Figure 1A,B, T84). In contrast, when the cells were pretreated with *S. boulardii* for 15 h (*S. b* (ON)) before LT intoxication, we observed a limited effect on the reorganization of the actin cytoskeleton (Figure 1C, T84). We verified that overnight treatment of cells with *S. boulardii* alone had no effect (Figure 1D, T84). Next, we tested whether *S. boulardii* protected primary human umbilical vein endothelial cells (HUVECs) against intoxication. As previously shown, the treatment of HUVECs with LT for 24 h induced a strong remodeling of the actin cytoskeleton into stress fibers (Figure 1A,B, HUVEC). A manual quantification of cells with modified cytoskeleton organization revealed that 20% of the LT-treated cells contained thick actin cables, compared with 5.4% in control cells. In keeping with the above observations in epithelial cells, we measured a significant decrease in stress fiber formation in HUVECs pretreated with *S. boulardii* for 15 h before LT intoxication (Figure 1C, HUVEC). We verified that 15 h of cell treatment with *S. boulardii* alone had no effect on the actin cytoskeleton (Figure 1D, HUVEC). Treatment of cells with *S. boulardii* limits the cytotoxic effects of LT on actin organization.

### 2.2. S. boulardii Prevents LT-Induced Loss in Permeability

**Figure 2:**
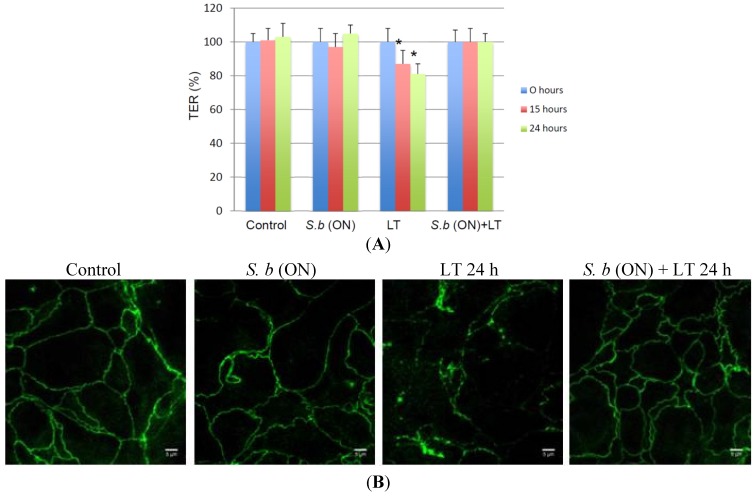
Protective effect of *S. boulardii* on LT-induced epithelium disruption. (**A**) Trans-epithelial resistance (TER) was measured at different time points in: control T84 monolayers (Control), T84 monolayers incubated overnight with *S. boulardii* alone (*S.b* (ON)), T84 monolayers incubated with LT alone (LT) and T84 monolayers incubated overnight with *S. boulardii* prior to the incubation with LT for 24 h (*S.b* (ON) + LT). TER values are displayed as percentages of initial values (*n* = 3). An asterisk denotes a significant difference *versus* control cells (*p* < 0.05, *n* = 3 independent experiments); (**B**) ZO-1 distribution was visualized in T84 monolayers control (control), incubated overnight with *S. boulardii* alone (*S.b* (ON)), monolayers incubated with LT alone for 24 h (LT 24 h), or monolayers incubated overnight with *S. boulardii* prior to the incubation with LT for 24 h (*S.b* (ON) + LT 24 h).

In endothelial cells, the reorganization of the actin cytoskeleton induced by LT treatment disrupts the monolayer, thereby increasing endothelial permeability [11,13,25]. To investigate the effect of LT on T84 permeability, we measured the trans-epithelial resistance (TER). As shown in Figure 2A, there was a significant decrease of 15%–20% in the TER 15–24 h after the addition of LT. After 24 h, the resistance decreased to 80%. To test a possible protective role of *S. boulardii* on the barrier function, we pretreated cells for 15 h with yeast cells. Under these conditions, we observed that pretreatment with *S. boulardii* significantly maintained the TER of LT-intoxicated monolayers compared with the control. An overnight challenge with *S. boulardii* as the sole treatment did not affect the TER (Figure 2A). In parallel, we investigated whether the LT-induced drop in the TER was associated with morphological modification of the tight junctions (TJs). Modification of TJ morphology was monitored by immunofluorescence staining with the zonula occludens-1 (ZO-1) marker. After 24 h of exposure to LT, T84 monolayers showed diffuse and non-linear staining patterns compared with the continuous ZO-1 staining pattern in untreated cells (Figure 2B). Interestingly, in cells pretreated with *S. boulardii* overnight, the continuous ZO-1 distribution was preserved (Figure 2B). An overnight challenge with *S. boulardii* as the sole treatment did not affect the ZO-1 staining pattern. Therefore, *S. boulardii* protects against the LT-induced disruption of the columnar epithelial cell barrier.

### 2.3. S. boulardii Induces Protective Effects on LT-Induced MEK-2 Cleavage

**Figure 3:**
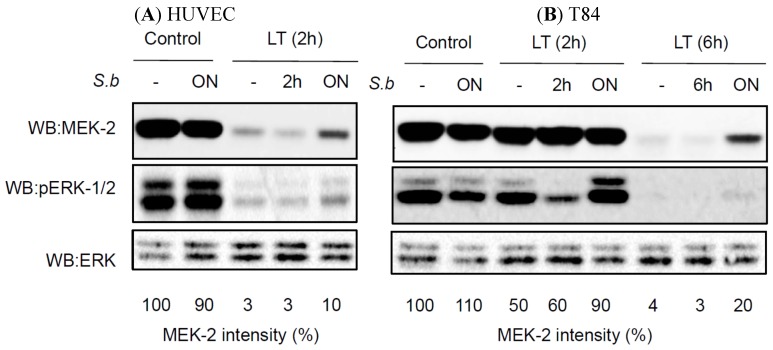
Protective effect of *S. boulardii* on LT-induced MEK2 cleavage. Western blot anti-MEK-2 and anti-phopho-ERK-1/2 (pERK-1/2) on cell extracts of HUVECs (**A**) and T84 (**B**). Cells were left untreated or treated with *S. b* 2 h, 6 h or overnight (ON) prior to treatment with LT for 2 or 6 h. Immunoblot ERK is shown as a loading control. Percentages display the decrease of MEK2 signal intensity in the different conditions, as compared to control cells. Western blots shown are representative of 5 independent experiments.

To understand the molecular mechanisms responsible for the prophylactic effect of *S. boulardii*, we analyzed MEK cleavage. Kinetic studies in HUVECs showed dramatic LT-induced cleavage of MEK-2 in as little as two hours after intoxication (the band intensity was decreased to 3% compared with the control) (Figure 3A). Notably, only pretreatment with *S. boulardii* was efficient and reduced MEK-2 cleavage by 30% compared with LT alone (Figure 3A). We next studied the enzymatic activity of LT in T84 cells. In these cells, the kinetics of MEK-2 cleavage was delayed compared to HUVECs: only 50% of MEK-2 was cleaved in T84 cells exposed for 2 h to LT, and 100% of MEK-2 cleavage occurred only 6 h after LT intoxication (Figure 3B). As expected from the TER analyses, overnight pretreatment with *S. boulardii* showed a protective effect against LT-induced MEK-2 cleavage (Figure 3B and Figure 2A). In T84 and HUVECs treated for 2 h with LT, we detected phospho-ERK only when the cells were co-treated with *S. boulardii* (Figure 3A). In both cell lines incubated concomitantly with *S. boulardii* and LT, we did not observe any effect of the yeast on LT-induced MEK-2 cleavage (Figure 3A,B). An overnight challenge with yeast alone had no effect on the MEK-2 levels in either HUVECs or T84 cells (Figure 3A,B).

### 2.4. S. boulardii Interacts with PA and LF

**Figure 4:**
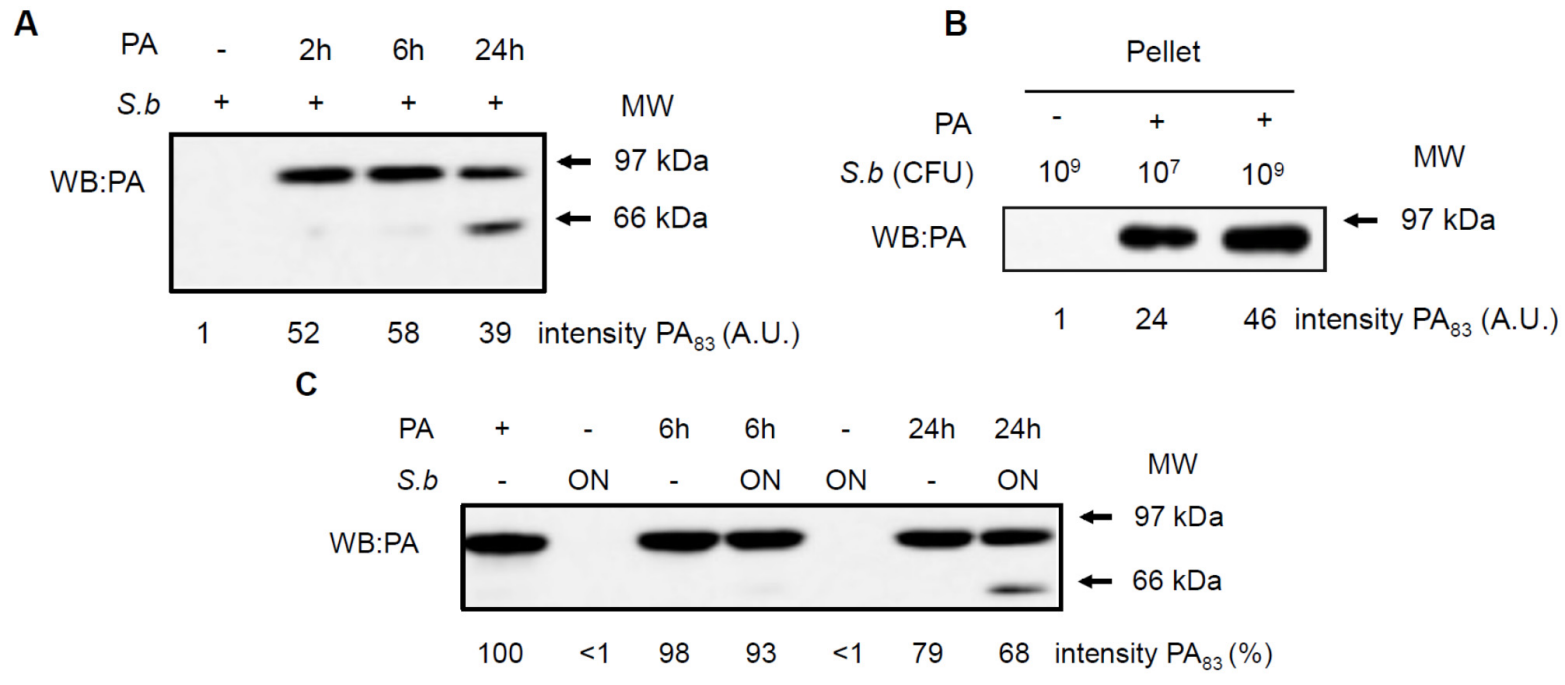
Impact of *S. boulardii* on the stability of protective antigen PA. Western blots anti-PA (WB: PA) show the co-precipitation of PA with *S. boulardii* after various time periods of co-incubations (**A**) or with different quantities of *S. boulardii* co-incubated 2 h (**B**); the cleavage of PA in the supernatant (**C**). Signal intensity were quantified by densitometric analysis and values are expressed as arbitrary units (**A**) and percentages as compared to PA alone (**C**). Western blots shown are representative of 5 independent experiments.

To investigate whether *S. boulardii* protects cells via a direct interaction with the LT subunits, PA or LF, we performed a series of co-incubation experiments. Yeast cells were incubated with PA or LF for different times before assessment of the amount of toxin co-precipitated with the yeast fraction or quantification of the toxin present in the culture supernatant (for details, see Material and Methods). Figure 4A shows the amount of PA proteins co-precipitated with the yeast cells after different co-incubation times: 2, 6 or 24 h. In the absence of *S. boulardii*, we did not detect PA in the pellet. A protein that was recognized by the anti-PA antibody and that migrated to the same distance as PA was present after 2, 6 and 24 h of co-incubation with *S. boulardii*. To investigate whether the adhesion of PA depends on the quantity of yeast, we performed an experiment in which the same quantity of PA was added to an exponentially grown yeast culture containing 10^7^ CFU/mL or to a stationary culture containing 10^9^ CFU/mL. Figure 4B shows that under both conditions, PA protein that co-precipitated with the yeast was detected and the amount of PA was correlated with the number of yeast cells. Figure 4C shows PA detection in the yeast supernatant (proteins not bound to the yeast). Notably, the PA factor was visualized as a single band of approximately 83 kDa, irrespective of the presence of *S. boulardii*, at all incubation time points. Moreover, a 24-hour co-incubation of PA with *S. boulardii* (last line) showed a supplementary band of lower molecular weight (approximately 63 kDa), probably corresponding to the cleaved form of PA.

**Figure 5:**
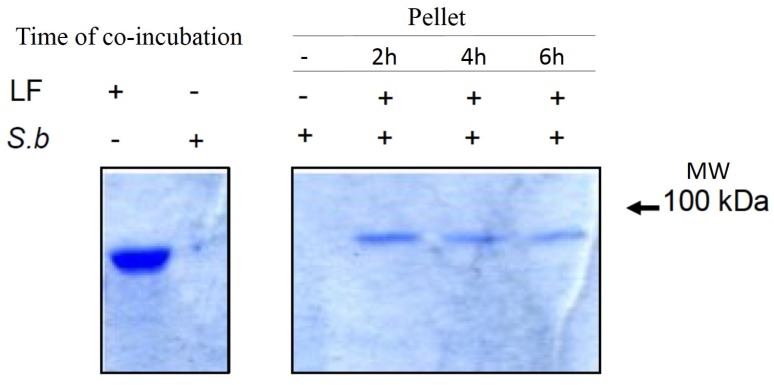
Co-precipitation of LF with *S. boulardii*. Co-precipitation of LF with *S. boulardii* after 2, 6 and 24 h of co-incubation. Proteins were resolved on SDS-PAGE 12% and visualized by Coomassie blue staining.

Furthermore, using a similar assay, we observed the co-precipitation of LF with the *S. boulardii* cell fraction (Figure 5).

## 3. Discussion

Currently available prophylactic and therapeutic options to combat anthrax are limited. The only approved treatment is protective antigen targeting with a monoclonal antibody (raxibacumab), which is mostly used to treat inhalational anthrax [26]. Better identification and validation at the molecular level of anthrax toxin therapeutic targets, as well as the elucidation of the parameters determining the corresponding therapeutic windows, are still necessary for more effective therapeutic options. The actual efforts focus mainly on (i) new vaccines; (ii) selective antimicrobial agents against *B. anthracis*; and (iii) effective anti-toxin compounds [13,27]. Data presented in this manuscript described the anti-toxin effect of the yeast probiotic *S. boulardii*, which is currently used in the treatment of gastro-intestinal infectious diseases.

Systemic dissemination is possible for each of the distinct forms of a clinical anthrax infection. In this regard, *B. anthracis* demonstrates an intrinsic ability to compromise a variety of epithelial and endothelial barriers that are present in mammalian organ systems. It is widely viewed that the anthrax toxin released by replicating *B. anthracis* contributes greatly to the systemic infection by disrupting the integrity and functionality of various barrier tissues. *In vivo* treatment of several animal species with the LT toxin leads to mortality [1,28]. Using a series of histological and biological assessments, Chen *et al.*, have extensively characterized the effects of LT on intestinal tissue [23]. This group demonstrated that LT causes a dose-dependent disruption of the integrity of the intestinal epithelium, characterized by mucosal erosion, ulceration and bleeding.

In endothelial and epithelial cells, LT alters the cellular barrier function by affecting the tight junction (TJ) and adherens junction (AJ) complexes that consist of transmembrane proteins linking adjacent cells to the actin cytoskeleton [12,29,30,31]. Data presented in this study clearly demonstrate that the pre-incubation of polarized T84 cell monolayers with *S. boulardii* preserves the barrier function of the T84 cell monolayers during LT intoxication and maintains the ZO-1 perijunctional distribution.

The effect of LT on the actin cytoskeleton is a relatively recent discovery. Most of the studies focused on endothelial and epithelial cells and on immune cells such as macrophages or neutrophils. After exposure to LT, human endothelium and lung epithelial cells demonstrate progressive mechanical stiffness, blebbing and endothelial barrier disruption [24,25,29]. These morphological changes correlate with the reorganization of the actin cytoskeleton, characterized by thick, parallel actin stress fibers [11,12,13]. Unconventional formation and stabilization of stress fibers occurred in the presence of LT over the course of 6–24 h. We confirmed these observations in the polarized T84 cells as well as in the HUVEC model, in which 20% of the intoxicated cells displayed actin cables. Interestingly, the number of cells with stress fibers significantly dropped after pretreatment with *S. boulardii*. Kinetic studies of LT-induced MEK-2 cleavage showed that cleavage occurred rapidly in the HUVECs at 2 h after incubation. MEK-2 cleavage was not observed in the T84 cells in this time frame. Rather, the cleavage of MEK-2 in these cells occurred after 6 h of exposure to the toxin. When both cell lines were incubated concomitantly with *S. boulardii* and LT, we did not observe any effect of the yeast on LT-induced MEK-2 cleavage. However, when both cell lines were exposed to the yeast before incubation with the LT toxin, MEK-2 cleavage was reduced in cells incubated with the yeast. Hence, the protective effect of *S. boulardii* against LT infection requires pre-incubation with the yeast. This observation is not surprising and can be explained by the release of proteases into the medium by the replicating yeast. This hypothesis is supported by the presence of active peptide in the yeast supernatant in metabolically active *S. boulardii* grown for at least 24–48 h [17].

We show in this study that *S. boulardii* acts on PA in two ways: (i) by adhesion of PA to the cell wall and (ii) by inducing its cleavage. Because PA pre-pore formation is necessary for LF or EF binding and translocation, the unregulated cleavage of PA and its binding to *S. boulardii* could dramatically interfere with toxin action. The cleavage of PA by *S. boulardii* requires the actions of a protease. Yeasts synthesize proteases that are implicated in protein maturation. For example, Kex-2 is a pro-hormone that processes a serine protease that is secreted by the budding yeast *Saccharomyces cerevisiae*. Interestingly, Kex-2 shares structural similarities with the bacterial protease subtilisin and mammalian furin [32]. In *Saccharomyces boulardii* CNCMI-745, Castaglioulo *et al.* [17] identified a 54-kDa serine protease that cleaves *C. difficile* toxins A and B. Thus, we hypothesize that this or another protease secreted by *S. boulardii* is implicated in the cleavage of PA. *S. boulardii*, in addition to promoting PA cleavage, can also bind to PA. This association did not depend on the growth stage of the yeast (*i.e.*, the exponential or stationary growth phase), but the quantity of PA that bound to the yeast depended on the number of yeast present. Cholera toxin (CT) is another A/B type toxin that adheres to the *S. boulardii* cell wall through the B subunit, leading to the internalization of the A subunit necessary for cAMP signaling and trehalase activation in yeast [20]. These authors proposed that *S. boulardii* expresses a receptor structurally and functionally similar to the enterocyte receptor for the CT—ganglioside receptor GM1. The molecule of *S. boulardii* that is implicated in PA adhesion remains to be determined.

Taken together, our data suggest that the two fundamental protective mechanisms of *S. boulardii* during an anthrax infection are the binding and hydrolysis of the anthrax toxins.

## 4. Material and Methods

### 4.1. Cell Lines and Growth Conditions

The T84 human colonic cell line was obtained from the European Collection of Animal Cell Cultures (Salisbury, England) and grown in a 1:1 mixture of Dulbecco-Vogt modified Eagle’s medium and Ham’s-F12 medium (DMEM/F12) supplemented with 50 μg/mL penicillin, 50 μg/mL streptomycin (Sigma, St Louis, MO, USA) and 4% foetal bovine serum (Invitrogen, Carlsbad, CA, USA) as previously described [21]. Human umbilical vein endothelial cells (HUVECs) were purchased from PromoCell (Heidelberg, Germany) and maintained in human endothelial SFM (Invitrogen) with 50 μg/mL penicillin, 50 μg/mL streptomycin, 20% foetal bovine serum, 20 ng/mL FGF (Invitrogen), 10 ng/mL EGF (Invitrogen) and 1 μg/mL heparin (Sigma) as previously described [25].

### 4.2. Microorganisms

Cultures of *S. boulardii* were inoculated from lyophilized yeast (strain CNCM I-745. Syn. HANSEN CBS5926, BIOCODEX, Gentilly, France) and grown overnight at 37 °C, with shaking in Halvorston minimal medium with 2% glucose. In the preventive protocol prior to incubation with the toxins, yeast was added to the cell culture monolayer at a ratio of 1 yeast/cell and grown overnight to a ratio of 10 yeasts/cell.

### 4.3. Toxins

Recombinant toxins (LF and PA) were synthesized as previously described [12]. The LT toxin was obtained with a mixture of PA toxin and LF toxin in a ratio of 3/1 with a final concentration of 0.3 μg/mL PA and 0.1 μg/mL LF.

### 4.4. Trans-Epithelial Resistance Measurements

T84 cells were grown on collagen-coated 0.33 cm^2^ porous filter membranes (3-mm-diameter pores, Costar) (Sigma). Trans-epithelial resistance (TER) was measured using a Millicell-ERS apparatus (Millipore, Darmstadt, Germany). Under these conditions high TER values (>1000 Ω cm^2^) were consistently obtained in monolayers 7 days after post-seeding. The toxins and yeast were added to the apical compartment of the filter-grown cells, and TER was measured at different time as indicated in the figure.

### 4.5. Interaction of Yeast with PA or LF

*S. boulardii* were grown as previously described, harvested by centrifugation and re-suspended in DMEM/F12 without serum. A yeast suspension (50 μL) containing 0.5 × 10^7^ CFU/well was added to a 12-well culture plate containing 450 μL of DMEM/F12 without serum and grown overnight. The following day, LF or PA were added to the wells. Wells containing PA or LF alone and a well containing *S. boulardii* alone were set up as controls. After 2, 6 and 24 h of incubation, we centrifuged (1500 rpm) a 100 μL sample from each well, added running buffer to the supernatant and applied the sample to an SDS gel to detect the proteins. To determine whether the toxins could bind to the yeast, the pellets containing the yeast were washed several times in phosphate buffer, and the pellets were lysed in running buffer and applied to an SDS gel. PA detection was performed by western blotting using an anti-PA antibody (GeneTex Inc., Irvine, CA, USA), and LF detection was performed after staining the gel with Coomassie blue.

### 4.6. Western Blotting

Intoxicated cells (T84 cells or HUVECs) were washed with cold PBS and scraped with lysis buffer (50 mM Tris-HCl pH 7.5, 150 mM NaCl, 1% NP40, 2 mM Na_3_VO_4_, 1 mM EDTA, 1 μM aprotinin, 25 μM leupeptin, 1 μM pepstatin, 1 mM AEBSF, 10 mM NaF, 5 mM NaPPi, 10 mM β-glycerophosphate). The lysate was sonicated and solubilized for 30 min at 4 °C and then centrifuged at 14,000 rpm for 20 min at 4 °C. The protein concentration of the supernatant was determined using Bio-Rad DC reagents (Bio-Rad, Marnes-la-Coquette, France). Equal amounts (50 μg) of whole cell lysates were subjected to a 12% SDS-polyacrylamide gel. The proteins were transferred onto a polyvinylidene fluoride membrane (PVDF Hybond-P, Amersham, GE Healthcare, Velizy-Villacoublay, France) and incubated overnight at 4 °C with anti-MEK-2-N20 (Santa Cruz Biotechnology, Inc., Heidelberg, Germany) or anti-PA (GeneTex Inc., Irvine, CA, USA) primary antibodies followed by HRP-conjugated anti-rabbit or anti-mouse secondary antibodies, respectively (New England Biolabs, Evry, France). The blots were developed using the Enhanced Chemiluminescence detection system (GE Healthcare Life Sciences, Little Chalfont, UK).

### 4.7. Immunofluorescence Analyses

Immunofluorescence studies were performed on cells fixed in 4% paraformaldehyde (Sigma). The actin cytoskeleton was labelled using 1 μg/mL TRITC-conjugated phalloidin (Sigma). Immunosignals were analyzed with the LSM510-Meta confocal microscope (Carl Zeiss, Marly le Roi, France) with a 63× magnification lens. Each picture represents the projection of six serial confocal sections.

### 4.8. Statistical Analysis

All experiments were repeated at least three times. The results are presented as the mean ± SEM. Statistical significance was determined by analysis of variance with the StatView program, followed by post hoc comparison with the Bonferroni and Dunn tests. The level of significance was *p* < 0.05.

## References

[1] Mock M., Fouet A. (2001). Anthrax. Annu. Rev. Microbiol..

[2] Beatty M.E., Ashford D.A., Griffin P.M., Tauxe R.V., Sobel J. (2003). Gastrointestinal anthrax: Review of the literature. Arch. Intern. Med..

[3] Abramova F.A., Grinberg L.M., Yampolskaya O.V., Walker D.H. (1993). Pathology of inhalational anthrax in 42 cases from the Sverdlovsk outbreak of 1979. Proc. Natl. Acad. Sci. USA.

[4] Sweeney D.A., Hicks C.W., Cui X., Li Y., Eichacker P.Q. (2011). Anthrax infection. Am. J. Respir. Crit. Care Med..

[5] Moayeri M., Leppla S.H. (2009). Cellular and systemic effects of anthrax lethal toxin and edema toxin. Mol. Aspects Med..

[6] Collier R.J., Young J.A. (2003). Anthrax toxin. Annu. Rev. Cell Dev. Biol..

[7] Trescos Y., Tournier J.N. (2012). Cytoskeleton as an emerging target of anthrax toxins. Toxins.

[8] Leppla S.H. (1982). Anthrax toxin edema factor: A bacterial adenylate cyclase that increases cyclic AMP concentrations of eukaryotic cells. Proc. Natl. Acad. Sci. USA.

[9] Duesbery N.S., Webb C.P., Leppla S.H., Gordon V.M., Klimpel K.R., Copeland T.D., Ahn N.G., Oskarsson M.K., Fukasawa K., Paull K.D. (1998). Proteolytic inactivation of MAP-kinase-kinase by anthrax lethal factor. Science.

[10] Chavarria-Smith J., Vance R.E. (2013). Direct proteolytic cleavage of NLRP1B is necessary and sufficient for inflammasome activation by anthrax lethal factor. PLoS Pathog..

[11] Warfel J.M., Steele A.D., D’Agnillo F. (2005). Anthrax lethal toxin induces endothelial barrier dysfunction. Am. J. Pathol..

[12] Rolando M., Stefani C., Flatau G., Auberger P., Mettouchi A., Mhlanga M., Rapp U., Galmiche A., Lemichez E. (2010). Transcriptome dysregulation by anthrax lethal toxin plays a key role in induction of human endothelial cell cytotoxicity. Cell. Microbiol..

[13] Rolando M., Stefani C., Doye A., Acosta M.I., Visvikis O., Yevick H.G., Buchrieser C., Mettouchi A., Bassereau P., Lemichez E. (2015). Contractile actin cables induced by *Bacillus anthracis* lethal toxin depend on the histone acetylation machinery. Cytoskeleton.

[14] Trescos Y., Tessier E., Rougeaux C., Goossens P.L., Tournier J.N. (2015). Micropatterned macrophage analysis reveals global cytoskeleton constraints induced by *Bacillus anthracis* edema toxin. Infect. Immun..

[15] McFarland L.V. (2010). Systematic review and meta-analysis of *Saccharomyces boulardii* in adult patients. World J. Gastroenterol..

[16] Kelesidis T., Pothoulakis C. (2012). Efficacy and safety of the probiotic *Saccharomyces boulardii* for the prevention and therapy of gastrointestinal disorders. Therap. Adv. Gastroenterol..

[17] Castagliuolo I., Riegler M.F., Valenick L., LaMont J.T., Pothoulakis C. (1999). *Saccharomyces boulardii* protease inhibits the effects of *Clostridium difficile* toxins A and B in human colonic mucosa. Infect. Immun..

[18] Pothoulakis C., Kelly C.P., Joshi M.A., Gao N., O'Keane C.J., Castagliuolo I., Lamont J.T. (1993). *Saccharomyces boulardii* inhibits *Clostridium difficile* toxin A binding and enterotoxicity in rat ileum. Gastroenterology.

[19] Buts J.P., Dekeyser N., Stilmant C., Delem E., Smets F., Sokal E. (2006). *Saccharomyces boulardii* produces in rat small intestine a novel protein phosphatase that inhibits *Escherichia coli* endotoxin by dephosphorylation. Pediatr. Res..

[20] Brandao R.L., Castro I.M., Bambirra E.A., Amaral S.C., Fietto L.G., Tropia M.J., Neves M.J., Dos Santos R.G., Gomes N.C., Nicoli J.R. (1998). Intracellular signal triggered by cholera toxin in *Saccharomyces boulardii* and *Saccharomyces cerevisiae*. Appl. Environ. Microbiol..

[21] Czerucka D., Rampal P. (1999). Effect of *Saccharomyces boulardii* on cAMP- and Ca^2+^ -dependent Cl- secretion in T84 cells. Dig. Dis. Sci..

[22] Czerucka D., Roux I., Rampal P. (1994). *Saccharomyces boulardii* inhibits secretagogue-mediated adenosine 3′,5′-cyclic monophosphate induction in intestinal cells. Gastroenterology.

[23] Chen X., Kokkotou E.G., Mustafa N., Bhaskar K.R., Sougioultzis S., O’Brien M., Pothoulakis C., Kelly C.P. (2006). *Saccharomyces boulardii* inhibits ERK1/2 mitogen-activated protein kinase activation both *in vitro* and *in vivo* and protects against *Clostridium difficile* toxin A-induced enteritis. J. Biol. Chem..

[24] Lehmann M., Noack D., Wood M., Perego M., Knaus U.G. (2009). Lung epithelial injury by *B. anthracis* lethal toxin is caused by MKK-dependent loss of cytoskeletal integrity. PLoS One.

[25] Rolando M., Munro P., Stefani C., Auberger P., Flatau G., Lemichez E. (2009). Injection of *Staphylococcus aureus* EDIN by the *Bacillus anthracis* protective antigen machinery induces vascular permeability. Infect. Immun..

[26] Huang B., Xie T., Rotstein D., Fang H., Frucht D.M. (2015). Passive Immunotherapy Protects against Enteric Invasion and Lethal Sepsis in a Murine Model of Gastrointestinal Anthrax. Toxins.

[27] Beitzinger C., Bronnhuber A., Duscha K., Riedl Z., Huber-Lang M., Benz R., Hajós G., Barth H. (2013). Designed azolopyridinium salts block protective antigen pores *in vitro* and protect cells from anthrax toxin. PLoS One.

[28] Liu S., Zhang Y., Moayeri M., Liu J., Crown D., Fattah R.J., Wein A.N., Yu Z.X., Finkel T., Leppla S.H. (2013). Key tissue targets responsible for anthrax-toxin-induced lethality. Nature.

[29] Chen S., Fang H., Xie T., Auth R.D., Patel N., Murray P.R., Snoy P.J., Frucht D.M. (2012). Anthrax lethal toxin disrupts intestinal barrier function and causes systemic infections with enteric bacteria. PLoS One.

[30] Guichard A., McGillivray S.M., Cruz-Moreno B., van Sorge N.M., Nizet V., Bier E. (2010). Anthrax toxins cooperatively inhibit endocytic recycling by the Rab11/Sec15 exocyst. Nature.

[31] Xie T., Auth R.D., Frucht D.M. (2011). The effects of anthrax lethal toxin on host barrier function. Toxins.

[32] Nakayama K. (1997). Furin: A mammalian subtilisin/Kex2p-like endoprotease involved in processing of a wide variety of precursor proteins. Biochem. J..

